# Parkinson’s research with inclusion, diversity and equity (PRIDE): examining Parkinson’s disease symptoms among LGBTQ+ and non-LGBTQ+ adults

**DOI:** 10.3389/fpubh.2026.1928319

**Published:** 2026-08-24

**Authors:** Jason Flatt, Brittany Klenczar-Castro, Sydney Kenney, Binita Adhikari, Krystal Kittle, Jennifer Pharr, Erin Rook, Emylia Terry

**Affiliations:** 1 Department of Social and Behavioral Health, School of Public Health, University of Nevada, Las Vegas, Las Vegas, NV, United States; 2 Department of Community Health Education, School of Public Health and Health Sciences, University of Massachusetts Amherst, Amherst, MA, United States; 3 Department of Environmental and Global Health, School of Public Health, University of Nevada, Las Vegas, Las Vegas, NV, United States

**Keywords:** discrimination, disparities, LGBTQ+, Parkinson’s disease, social determinants of health

## Abstract

**Introduction:**

Nearly four million LGBTQ+ adults aged 50 and older live in the U.S., with up to 40,000 currently living with Parkinson’s disease (PD). However, little is known about PD-related symptoms in this population.

**Methods:**

From 2022 to 2025, we conducted a national cross-sectional study using a convenience sample of LGBTQ+ and non-LGBTQ+ individuals living with PD (*n* = 254). We administered validated PD and health-related measures to examine physical, mental and social health symptoms. Bivariate and regression models examined differences in symptom burden between LGBTQ+ and non-LGBTQ+ participants, as well as associations with sociodemographic characteristics and social determinants of health.

**Results:**

The sample included 89 LGBTQ+ and 165 non-LGBTQ+ individuals living with PD. On average, LGBTQ+ participants were 5.3 years younger than their non-LGBTQ+ counterparts (mean age: 62.9 vs. 68.3). Compared with non-LGBTQ+ participants, LGBTQ+ individuals living with PD were more likely to report anxiety (53.9% vs. 33.3%, *p* = 0.002), moderate-to-severe motor impairments (32.2% vs. 19.4% *p* = 0.024), and loneliness (18.8% vs. 8.5%, *p* = 0.037). Regarding social determinants of health, younger age, identifying as a person of color, and experiencing healthcare discrimination were associated with higher odds of reporting moderate-to-severe PD-related symptoms.

**Discussion:**

This study highlights potential disparities in PD-related symptoms, with LGBTQ+ individuals, persons of color, and those experiencing healthcare discrimination being more likely to report physical, mental and social health challenges. There is a need for further research on the health needs of diverse populations living with PD, including LGBTQ+ adults, as well as the development of tailored and inclusive healthcare strategies.

## 1 Introduction

Parkinson’s disease (PD) is the second most common neurodegenerative disorder worldwide, affecting over 6 million people globally, with prevalence projected to more than double by 2050 (1, 2). Although PD is often characterized by motor symptoms such as tremors, rigidity, and gait impairment, non-motor symptoms, including depression, anxiety, sleep disturbances, fatigue, and autonomic dysfunction, are also common and often contribute to disability and reduced quality of life (3, 4). Many individuals living with PD identify non-motor symptoms among their top concerns, highlighting the need to understand both non-motor and motor symptom experiences.

Beyond PD-related symptoms, many individuals live with multiple chronic health conditions that can complicate disease management and symptom progression. Comorbidities, such as depression and cardiovascular disease, have been associated with worse motor outcomes, greater cognitive impairment, and increased disability among individuals living with PD (5–7). These findings highlight that PD symptom burden is closely linked to both physical and mental health conditions, which may also influence disease progression and quality of life.

The experiences of individuals living with PD are further shaped by social determinants of health, including economic stability, healthcare access, social relationships, and experiences of discrimination. Structural inequities contribute to higher rates of chronic illness and poorer health outcomes among historically marginalized populations, including racial and ethnic minorities, LGBTQ+ individuals, people from lower socioeconomic backgrounds, and those living in rural communities (8, 9). These determinants may create additional barriers to managing PD symptoms, accessing specialty care, and maintaining overall wellbeing.

Recent studies suggests that LGBTQ+ individuals living with PD may experience unique challenges related to symptom burden and healthcare access. For instance, studies find that LGBTQ+ individuals with PD report an earlier age of diagnosis, greater disease burden, and more frequent experiences of healthcare discrimination compared with their cisgender, heterosexual counterparts (10, 11). These compounding factors often contribute to worse quality of life for LGBTQ+ individuals living with PD.

Improving understanding of symptom experiences among diverse populations is critical for developing more equitable and person-centered approaches to PD care. Despite growing recognition of health disparities among diverse aging populations, relatively little research has examined whether PD symptom burden differs by LGBTQ+ identity. The present study seeks to examine differences in physical, mental and social health symptoms among LGBTQ+ and non-LGBTQ+ individuals living with PD, and to explore social determinants associated with these symptoms.

## 2 Methods

### 2.1 Study design

From 2022 to 2025, we conducted an observational, cross-sectional study with a convenience sample of LGBTQ+ and non-LGBTQ+ individuals living with PD and/or their family caregiving via a telephone survey. Participant inclusion criteria were: (1) identified as LGBTQ+ or non-LGBTQ+, (2) 18+ years of age, and (3) had the ability to complete the phone or online survey in English or Spanish. The telephone surveys were developed using computer-assisted telephone interviewing, and trained interviewer conducted the survey by telephone. The one-time survey took up to 45 min to complete. Of the 254 completed surveys, 187 were completed directly by individuals living with PD, whereas 67 were completed by family caregivers serving as proxy respondents on behalf of the individual living with PD. In many of these interviews, the individual living with PD was present during the telephone survey and contributed to the responses.

Participants were recruited via print and social media (e.g., The Advocate), through communications with local and national LGBTQ+ organizations (e.g., SAGE, Center Link, LGBTQ+ Centers, Pride Festivals), local and national organizations (e.g., Michael J. Fox Foundation’s Buddy Network, ClinicalTrials.gov, Fox Trial Finder), senior centers, PD support groups and/or exercise groups, webinars, and by asking healthcare providers to distribute the study flyer. Participants received $25 for survey completion. The survey collected demographic information, including age, sexual orientation, gender identity, race/ethnicity, education, household income, living arrangements, and healthcare access. Demographic characteristics were self-reported, including age, gender identity (man, woman, transgender men, or intersex), and sexual orientation (heterosexual, gay or lesbian, bisexual, queer, or questioning), race (white, black, Asian, American Indian/Native Alaskan, and other), ethnicity (Hispanic or Latino), education (<high school versus some college and bachelor degree or higher), income level (less than $20,000–100,000) and marital status (married or partnered vs. nor married or partnered). To measure health status, participants self-rated their health and reported their experiences with chronic conditions, mental health, relevant health diagnoses, psychological symptoms, healthcare satisfaction, and experiences with healthcare discrimination. Existing scales and validated measures previously tested with caregivers, individuals living with PD, and/or LGBTQ+ populations were used.

### 2.2 Parkinson’s disease symptoms

To examine motor and non-motor PD symptoms, the Movement Disorder Society-sponsored revision of the Unified Parkinson’s Disease Rating Scale (MDS-UPDRS), widely used and validated measure of PD symptoms (12, 13). We utilized Part I (Non-Motor Experiences of Daily Living) and Part II (Motor Experiences of Daily Living) of the MDS-UPDR (13). The MDS-UPDR questions are rated on a 4-point Likert scale (0 = normal, 1 = slight, 2 = mild, 3 = moderate, 4 = severe), can be administered to incorporate the person living with PD and/or the caregiver (13, 14). For Part I (non-motor symptoms), scores ranged from 0 to 56, with those who scored 0–14 classified as having mild non-motor symptoms, 15–30 as moderate non-motor symptoms, and 31–56 as severe non-motor symptoms. For Part II (motor symptoms), the scores ranged from 0 to 52, with those who scored 0–13 classified as having mild motor symptoms, 14–26 as moderate motor symptoms, and 27–52 as severe motor symptoms (13). The scales have demonstrated good internal consistency, comprehensively assesses both motor and non-motor PD symptoms. Additionally, higher scores on the MDS-UPDRS Parts I and II reflect greater disease severity and disability, which have been found to be associated with worse quality of life for individuals living with PD and increased caregiver burden (15–17).

#### 2.2.1 Mental health outcomes

Depressive symptoms were assessed using the Patient Health Questionnaire-9 (PHQ-9), a widely used and validated self-report measure of depression severity in both general and chronic illness populations, including PD. PHQ-9 consists of nine items corresponding to symptoms of depression, and participants reported how frequently they had experienced each symptom over the past 2 weeks using a 4-point Likert scale ranging from 0 (not at all) to 3 (nearly every day) (18). Total scores were interpreted using established cut-off scores 9 or higher (depressed) vs. <9 (not depressed) (19). Depression represents one of the most common and impactful non-motor symptoms of PD, with greater PD severity associated with worse depressive outcomes (15–17).

#### 2.2.2 Social health outcomes

Social health outcomes included loneliness, assessed with the UCLA Loneliness Scale, and health-related quality of life measured using the Parkinson’s Disease Questionnaire-8 (PDQ-8). We utilized the 3-item UCLA Loneliness short scale, which is rated on a 3-point Likert scale (Hardly ever = 1, some of the time = 2 points, and often = 3) with a total score ranging from 3 to 9, with higher scores indicating greater feelings of loneliness, and categorized as <5 not lonely and 6–9 lonely (20). The PDQ-8 is a validated, patient-reported measure specifically designed to capture the impact of PD on multiple domains of quality of life, including mobility, activities of daily living, emotional wellbeing, stigma, social support, cognition, communication, and bodily discomfort. PDQ-8 was scored with a maximum score of 32; those who scored 0–12 were classified as having mild quality of life impairments, 13–32 moderate-to-severe quality of life impairments (21). Discrimination in healthcare exposures was reported using seven questions related to denial or poor-quality care, healthcare avoidance due to mistreatment, provider refusal of care, harassment, and the need to educate providers about one’s sexual orientation and/or gender identity (22). Discrimination exposures were categorized as individuals experiencing any of the seven discrimination experiences reported vs. individuals experiencing none of the discrimination experiences.

### 2.3 Statistical analysis

All analyzes were conducted using SPSS Version 30 (Armonk, NY: IBM Corp). Results were provided as mean ± standard deviation for continuous variables, and frequency counts and percentages for categorical variables. Group differences between LGBTQ+ and non-LGBTQ+ participants living with PD were examined using Pearson chi-square tests for categorical variables and independent-samples *t*-tests for continuous variables (e.g., age). Fisher’s Exact Test was applied when expected cell counts fell below five.

To examine associations between sociodemographic characteristics and health outcomes, a series of bivariate chi-square analyzes was performed. Health outcomes included PD non-motor and motor symptoms, loneliness, health-related quality of life, and depressive symptoms. Sociodemographic predictors included age (dichotomized as <65 vs. ≥65). Age was collected as a continuous variable and summarized descriptively using four categories (20–49, 50–64, 65–79, and ≥80 years) in Table 1. For bivariate and multivariable analyzes, age was dichotomized as <65 vs. ≥65 years based on the conventional older adult threshold used in aging research, race (white vs. people of color), LGBTQ+ identity, marital status (married vs. not married), educational attainment, and household income. Experiences of healthcare discrimination were also examined as correlates of health outcomes using chi-square tests.

**Table 1:** Sociodemographic characteristics by LGBTQ+ status, *N* = 254.

Characteristic	LGBTQ+ (*n* = 89)		Non-LGBTQ+ (*n* = 165)		Total		*p*
	*n*	%	*n*	%	*n*	%	
Age (years), M (SD)	62.93 (15.96)		68.26 (15.14)		66.39 (15.49)		0.004
Age categories (years)							0.041
20–49	10	11.2%	10	6.1%	20	7.9%	
50–64	27	30.3%	39	23.8%	66	26.1%	
65–79	48	53.9%	91	55.5%	139	54.9%	
80+	4	4.5%	24	14.6%	28	11.1%	
Sex at birth							0.017
Male	59	66.3%	83	50.3%	142	55.9%	
Female	30	33.7%	82	49.7%	112	44.1%	
Gender identity							–^a^
Man	57	64.0%	107	64.8%	164	64.6%	–^a^
Woman	27	30.3%	57	34.5%	84	33.1%	–^a^
Transgender man	1	1.1%	0	0.0%	1	0.4%	–^a^
Transgender woman	2	2.2%	0	0.0%	2	0.8%	–^a^
Non-binary/genderqueer/GNC	2	2.2%	0	0.0%	2	0.8%	–^a^
Ethnicity, Hispanic/Latino	6	6.8%	13	8.0%	19	7.6%	0.741
Race							0.694
Asian	0	0.0%	3	1.8%	3	1.2%	
Black or African American	13	14.6%	23	14.0%	36	14.2%	
White	71	79.8%	131	79.0%	202	79.8%	
Another race	2	2.2%	4	2.4%	6	2.4%	
Multiracial	3	3.4%	3	1.8%	6	2.4%	
Sexual orientation							–^a^
Asexual	6	6.7%	0	0.0%	6	2.3%	–^a^
Bisexual	17	19.2%	0	0.0%	17	6.6%	–^a^
Gay	49	55.0%	0	0.0%	49	19.2%	–^a^
Lesbian	14	15.8%	0	0.0%	14	5.5%	–^a^
Queer	3	3.3%	0	0.0%	3	1.5%	–^a^
Heterosexual	0	0.0%	165	100.0%	165	64.9%	–^a^
Education							0.683
High school or less	8	9.1%	16	9.8%	24	9.5%	
Some college/associate degree	14	15.9%	33	20.1%	47	18.7%	
Bachelor’s degree or higher	66	75.0%	115	70.1%	181	71.8%	
Income level							0.209
Less than $20,000	5	6.0%	8	5.6%	13	5.7%	
$20,001–$40,000	22	26.5%	25	17.4%	47	20.7%	
$40,001–$60,000	8	9.6%	24	16.7%	32	14.1%	
$60,001–$80,000	12	14.5%	28	19.4%	40	17.6%	
$80,001–$100,000	4	4.8%	14	9.7%	18	7.9%	
$100,000+	32	38.6%	45	31.3%	77	33.9%	
Marital status							0.052
Married or partnered	51	57.3%	113	68.5%	164	64.6%	
Not married or partnered	38	42.7%	52	31.5%	90	35.4%	

–^a^ = Chi-square analysis not conducted due to insufficient cell size (expected cell count <5). M, mean; SD, standard deviation. Bold *p*-values indicate statistical significance (*p* < 0.05).

Multivariate logistic regression models were conducted to identify social determinants associated with the physical, social, and mental health of individuals living with PD. Only variables that were significantly associated in bivariate analyzes were tested in the final regression model. Separate models were estimated for each outcome: non-motor PD symptoms (mild vs. moderate-to-severe), motor symptoms (mild vs. moderate-to-severe), loneliness (not lonely vs. lonely), quality of life (mild vs. moderate-to-severe impairment in quality of life), and depressive symptoms (mild vs. moderate-to-severe depression symptoms). Predictors were entered simultaneously into each model, including age, race/ethnicity, LGBTQ+ identity, marital status, income level, and experiencing healthcare discrimination. Model fit was evaluated using the Nagelkerke *R*^2^ statistic, with results presented as adjusted odds ratios (AORs) and 95% confidence intervals (CIs). Sample sizes varied across outcomes due to item-level non-response (MDS Part I, *n* = 251; MDS Part II, *n* = 247; PDQ-8, *n* = 253, of *N* = 254 total). The larger reduction in sample size for the PHQ-9 (*n* = 187) and UCLA Loneliness Scale (*n* = 187) reflects the fact that these two measures assess internal psychological states (depressive symptoms and subjective loneliness) and were administered only to individuals living with PD who completed the interview directly; they were not administered to proxy respondents (caregivers) reporting on behalf of a person with PD, as proxies cannot reliably report on another individual’s internal subjective experiences. Bivariate chi-square analyzes used all available non-missing data for each specific comparison (available-case/pairwise analysis). For the multivariable logistic regression models, listwise (complete-case) deletion was applied across the outcome and all six predictor variables simultaneously, yielding a consistent analytic sample of *n* = 171 across all five models. All tests were two-tailed, and statistical significance was set at *p* < 0.05. We did not adjust for multiple comparisons given the exploratory focus of our study.

## 3 Results

### 3.1 Sociodemographic characteristics

A total of 254 individuals living with PD were included, of whom 89 (35.0%) identified as LGBTQ+ and 165 (65.0%) as non-LGBTQ+ (Table 1). The mean age of participants differed significantly between groups, with LGBTQ+ individuals being younger on average compared with non-LGBTQ+ individuals (62.93 vs. 68.26 years, *p* = 0.004). The age distribution also varied significantly, with a higher proportion of LGBTQ+ participants in the younger age categories (20–49 years: 11.2% vs. 6.1%), while non-LGBTQ+ participants were more represented in the oldest age group (≥80 years: 14.6% vs. 4.5%, *p* = 0.041).

Sex assigned at birth differed significantly between groups (*p* = 0.017), with a higher proportion of males among LGBTQ+ participants (66.3%) compared with non-LGBTQ+ participants (50.3%). However, there were no statistically significant differences across the gender identity categories. The majority of participants identified as men (64.6%) or women (33.1%), with a smaller proportion (1.9%) identifying as transgender or non-binary (*n* = 5). No significant differences were observed between LGBTQ+ and non-LGBTQ+ participants in terms of race/ethnicity. There were also no significant differences in educational attainment, with the majority holding a bachelor’s degree or higher (75.0% LGBTQ+ vs. 70.1% non-LGBTQ+). Similarly, income distribution did not differ significantly between groups, although a higher proportion of LGBTQ+ participants reported incomes above $100,000 (38.6% vs. 31.3%). There were marginal differences in marital status between groups (*p* = 0.052), with LGBTQ+ participants being less likely to be married or partnered (57.3% vs. 68.5%).

### 3.2 Health outcomes

Next, we examined differences in health among LGBTQ+ and non-LGBTQ+ individuals living with PD. Among LGBTQ+ individuals living with PD, we found a higher prevalence of anxiety, HIV, and mental health conditions (Table 2). Exploring the MDS-Part 1 (non-motor symptoms) and Part II (motor symptoms), we found that LGBTQ+ individuals living with PD were more likely to report moderate-to-severe motor impairments compared with non-LGBTQ+ individuals living with PD (32.2% vs. 19.4%, *p* = 0.02). LGBTQ+ individuals living with PD also reported greater loneliness than non-LGBTQ+ individuals living with PD (18.8% vs. 8.5%, *p* = 0.037). However, there were no significant differences in quality of life or depressive symptoms between LGBTQ+ and non-LGBTQ+ individuals living with PD.

**Table 2:** Prevalence of health outcomes by LGBTQ+ status.

Health conditions	LGBTQ+ (*n* = 89)		Non-LGBTQ+ (*n* = 165)		Total		*p*
	*n*	%	*n*	%	*n*	%	
HTN	36	40.4%	70	42.4%	106	41.7%	0.791
Heart attack	10	11.2%	8	4.8%	18	7.1%	0.073
Osteoporosis	18	20.2%	22	13.3%	40	15.7%	0.154
Angina/CHD	6	6.7%	18	10.9%	24	9.4%	0.279
Depression	40	44.9%	62	37.6%	102	40.2%	0.284
Stroke	6	6.7%	9	5.5%	15	5.9%	0.781
Anxiety	48	53.9%	55	33.3%	103	40.6%	**0.002**
Arthritis	35	39.3%	58	35.2%	93	36.6%	0.510
Asthma	12	13.5%	19	11.5%	31	12.2%	0.648
PTSD	10	11.2%	16	9.7%	26	10.25%	0.699
Cancer	24	27%	57	34.5%	81	31.9%	0.259
Kidney disease	5	5.6%	7	4.2%	12	4.7%	0.758
Diabetes	15	16.9%	28	17%	43	16.9%	0.981
COPD	7	7.9%	9	5.5%	16	6.3%	0.589
HIV	6	6.7%	0	0%	6	2.4%	**0.002**
Cardiovascular	41	46.1%	86	52.1%	127	50%	0.357
Mental health	58	65.2%	77	46.7%	135	53.1%	**0.005**
Respiratory	17	19.1%	26	15.8%	43	16.9%	0.498
Any musculoskeletal	42	47.2%	73	44.2%	115	45.3%	0.652
Comorbidity							
No comorbidity	6	6.7%	17	10.3%	23	9.1%	0.345
≥1 comorbidities	83	93.3%	148	89.7%	231	90.9%	
MDS Part I [non-motor aspects of experiences of daily living (nM-EDL)]							
Mild	52	59.1%	112	68.7%	164	65.3%	0.126
Moderate to severe	36	40.9%	51	31.3%	87	34.7%	
MDS Part II [motor aspects of experiences of daily living (M-EDL)]							
Mild	59	67.8%	129	80.6%	188	76.1%	**0.024**
Moderate to severe	28	32.2%	31	19.4%	59	23.9%	
Quality of life							
Mild Impairment	64	71.9%	121	73.8%	185	73.1%	0.749
Moderate to severe	25	28.1%	43	26.2%	68	26.9%	
Depressive symptoms							
Minimal-mild	50	72.5%	93	78.8%	143	76.5%	0.323
Moderate–severe	19	27.5%	25	21.2%	44	23.5%	
Loneliness							
Not Lonely	56	81.2%	108	91.5%	164	87.7%	**0.037**
Lonely	13	18.8%	10	8.5%	23	12.3%	

AOR, adjusted odds ratio; CI, confidence interval. Bold *p*-values indicate statistical significance (*p* < 0.05).

### 3.3 Sociodemographic characteristics, healthcare discrimination, and PD-related health outcomes

Next, we examined bivariate associations between sociodemographic factors and healthcare discrimination with PD-related physical, mental, and social health outcomes. We found that age, race/ethnicity, income, marital status, and reporting healthcare discrimination were associated with moderate-to-severe PD-related symptoms (Table 3), including the non-motor and motor impairments, loneliness, quality of life, and depression (all *p*-values < 0.05).

**Table 3:** Bivariate associations between demographic characteristics, healthcare discrimination, and health outcomes.

Demographic	Subgroup	Non-motor symptoms (MDS Part I), *N* = 251		*p*	Motor symptoms (MDS Part II), *N* = 247		*p*	Loneliness (UCLA), *N* = 187		*p*	Quality of life (PDQ-8), *N* = 253		*p*	Depressive symptoms (PHQ-9), *N* = 187		*p*
		Mild	Mod–severe		Mild	Mod–severe		Not lonely	Lonely		Mild	Mod–severe		Min–mild	Mod–severe	
Age	<65 years	42 (25.6%)	44 (50.6%)	<0.001^***^	57 (30.3%)	29 (49.2%)	0.008^**^	49 (35.5%)	20 (40.8%)	0.58	56 (30.3%)	30 (44.1%)	0.039^*^	40 (28.0%)	29 (65.9%)	<0.001^***^
	≥65 years	122 (74.4%)	43 (49.4%)		131 (69.7%)	30 (50.8%)		89 (64.5%)	29 (59.2%)		129 (69.7%)	38 (55.9%)		103 (72.0%)	15 (34.1%)	
Race	White	135 (82.3%)	66 (75.9%)	0.223	162 (86.2%)	37 (62.7%)	<0.001^***^	128 (92.8%)	35 (71.4%)	<0.001^***^	161 (87.0%)	41 (60.3%)	<0.001^***^	132 (92.3%)	31 (70.5%)	<0.001^***^
	People of color	29 (17.7%)	21 (24.1%)		26 (13.8%)	22 (37.3%)		10 (7.2%)	14 (28.6%)		24 (13.0%)	27 (39.7%)		11 (7.7%)	13 (29.5%)	
LGBTQ+ status	LGBTQ+	52 (31.7%)	36 (41.4%)	0.126	59 (31.4%)	28 (47.5%)	0.024^*^	45 (32.6%)	24 (49.0%)	0.749	64 (34.6%)	25 (36.8%)	0.041^*^	50 (35.0%)	19 (43.2%)	0.323
	Non-LGBTQ+	112 (68.3%)	51 (58.6%)		129 (68.6%)	31 (52.5%)		93 (67.4%)	25 (51.0%)		121 (65.4%)	43 (63.2%)		93 (65.0%)	25 (56.8%)	
Marital status	Married	114 (69.5%)	49 (56.3%)	0.037^*^	127 (67.6%)	32 (54.2%)	0.062	101 (73.2%)	17 (34.7%)	<0.0001^***^	127 (68.6%)	37 (54.4%)	0.036^*^	100 (69.6%)	18 (40.9%)	<0.001^***^
	Not married	50 (30.5%)	38 (43.7%)		61 (32.4%)	27 (45.8%)		37 (26.8%)	32 (65.3%)		58 (31.4%)	31 (45.6%)		43 (30.1%)	26 (59.1%)	
Education	<College degree	41 (25.2%)	28 (32.2%)	0.236	52 (27.7%)	17 (29.3%)	0.807	34 (24.6%)	15 (30.6%)	0.414	49 (26.6%)	22 (32.4%)	0.037^*^	34 (23.8%)	15 (34.1%)	0.174
	College degree+	122 (74.8%)	59 (67.8%)		136 (72.3%)	41 (70.7%)		104 (75.4%)	34 (69.4%)		135 (73.4%)	46 (67.6%)		109 (76.2%)	29 (65.9%)	
Income	<$40,000	29 (20.4%)	29 (34.9%)	0.016^*^	41 (24.6%)	18 (32.7%)	0.234	20 (16.0%)	27 (58.7%)	<0.001^***^	36 (21.7%)	24 (39.3%)	0.007^**^	27 (21.1%)	20 (46.5%)	0.005^**^
	≥$40,000	113 (79.6%)	54 (65.1%)		126 (75.4%)	37 (67.3%)		105 (84.0%)	19 (41.3%)		130 (78.3%)	37 (60.7%)		101 (78.9%)	23 (53.5%)	
Healthcare discrimination	None	52 (46.4%)	20 (27.0%)	0.008^**^	62 (44.6%)	10 (21.3%)	0.005^**^	68 (49.6%)	4 (8.2%)	<0.001^***^	69 (46.9%)	3 (7.7%)	<0.001^***^	65 (45.8%)	7 (15.9%)	<0.001^***^
	Any discrimination	60 (53.6%)	54 (73.0%)		77 (55.4%)	37 (78.7%)		69 (50.4%)	45 (91.8%)		78 (53.1%)	36 (92.3%)		77 (54.2%)	37 (84.1%)	

Values are *n* (%). Percentages reflect the distribution of each outcome category across demographic subgroups (column percentages), consistent with the original analysis. *p*-values are from Pearson’s chi-square test (Fisher’s exact test used where expected cell counts were <5). MDS, Movement Disorder Society Unified Parkinson’s Disease Rating Scale; PDQ-8, Parkinson’s Disease Questionnaire-8; PHQ-9, Patient Health Questionnaire-9; UCLA, UCLA Loneliness Scale. ^*^p < 0.05; ^**^p < 0.01; ^***^p < 0.001.

In our adjusted regression models (Table 4), younger age, identifying as a person of color, and experiencing healthcare discrimination emerged as the most consistent factors associated with moderate-to-severe PD-related symptoms. For non-motor symptoms, younger age (<65 years) was associated with more than three times the odds of reporting moderate-to-severe symptoms compared to those aged 65 and older (AOR = 3.39, 95% CI = 1.66–6.93, *p* < 0.001), after adjusting for other sociodemographic factors and healthcare discrimination. People of color living with PD had more than five times the odds of reporting moderate-to-severe motor symptoms (AOR = 5.06, 95% CI = 1.78–14.39, *p* = 0.002). For loneliness, people of color living with PD (AOR = 3.87, 95% CI = 1.23–12.15, *p* = 0.020), those who were not married (AOR = 2.69, 95% CI = 1.02–7.05, *p* = 0.044), individuals with an income less than $40,000 (AOR = 5.21, 95% CI = 1.93–14.04, *p* < 0.001), and those who experienced healthcare discrimination (AOR = 16.19, 95% CI = 4.25–61.63, *p* < 0.001) had higher odds of loneliness. For quality of life, younger age (AOR = 3.27, 95% CI = 1.32–7.84, *p* = 0.010), people of color (AOR = 3.50, 95% CI = 1.15–10.30, *p* = 0.027), and experiencing healthcare discrimination (AOR = 7.90, 95% CI = 2.10–29.00, *p* = 0.002) were associated with greater odds of reporting moderate-to-severe impairments in PD-related quality of life. Finally, younger age (AOR = 4.64, 95% CI = 1.90–11.20, *p* < 0.001), not being married (AOR = 2.60, 95% CI = 1.02–6.64, *p* = 0.045), and experiencing healthcare discrimination (AOR = 4.17, 95% CI = 1.44–12.08, *p* = 0.008) were associated with higher odds of reporting moderate-to-high depressive symptoms (Table 4).

**Table 4:** Multivariate logistic regression models examining social determinants of health outcomes among individuals living with Parkinson’s disease.

Predictor	Non-motor symptoms			Motor symptoms			Loneliness			Quality of life			Depressive symptoms		
	AOR	95% CI	*p*	AOR	95% CI	*p*	AOR	95% CI	*p*	AOR	95% CI	*p*	AOR	95% CI	*p*
Age (Ref: ≥65 years) <65 years	3.39	1.66–6.93	<0.001^***^	1.56	0.72–3.43	0.263	0.83	0.33–2.11	0.702	3.27	1.32–7.84	0.010^*^	4.64	1.90–11.20	<0.001^***^
Race (Ref: white) People of color	1.70	0.61–4.71	0.319	5.06	1.78–14.39	0.002^**^	3.87	1.23–12.15	0.020^*^	3.50	1.15–10.60	0.027^*^	2.30	0.76–6.80	0.138
LGBTQ+ (ref: non-LGBTQ+) LGBTQ+	0.88	0.43–1.80	0.729	0.62	0.28–1.36	0.239	1.30	0.54–3.15	0.555	1.45	0.61–3.50	0.385	1.40	0.59–3.30	0.432
Marital status (ref: married) Not married	1.32	0.61–2.87	0.472	1.48	0.63–3.50	0.370	2.69	1.02–7.05	0.044^*^	0.94	0.35–2.60	0.919	2.60	1.02–6.64	0.045^*^
Income (ref: >$40,000) <$40,000	1.73	0.75–4.00	0.197	0.73	0.28–1.88	0.515	5.21	1.93–14.04	<0.001^***^	2.80	0.94–7.70	0.062	2.70	0.98–7.07	0.055
Healthcare discrimination (ref: none) Any discrimination	1.63	0.76–3.48	0.209	2.25	0.89–5.67	0.084	16.19	4.25–61.63	<0.001^***^	7.90	2.10–29.00	0.002^**^	4.17	1.44–12.08	0.008^**^
Model *χ*^2^ (df)	26.29 (6)^***^			25.01 (6)^***^			65.75 (6)^***^			40.95 (6)^***^			47.61 (6)^***^		
Cox & Snell *R*^2^	0.142			0.136			0.319			0.213			0.243		
Nagelkerke *R*^2^	0.192			0.199			0.464			0.329			0.359		

AOR, adjusted odds ratio; CI, confidence interval. All predictors entered simultaneously in each model (*N* = 171 per model; listwise deletion for missing predictor data). Model *χ*^2^, omnibus likelihood-ratio test of the full model against the intercept-only model (df = 6 for all models). Cox & Snell *R*^2^ and Nagelkerke *R*^2^ are reported as measures of model fit, as specified in the methods (data analysis). ^*^p < 0.05; ^**^p < 0.01; ^***^p < 0.001.

## 4 Discussion

### 4.1 Health disparities among persons living with Parkinson’s disease

When examining several social determinants of health, younger age, identifying as a person of color, and experiencing healthcare discrimination emerged as the most consistent predictors of poorer physical, mental and social health outcomes. Within this broader context, LGBTQ+ individuals living with PD reported a higher burden of health and social challenges, including more severe motor impairment and loneliness, than their non-LGBTQ+ counterparts. LGBTQ+ individuals living with PD also experienced a greater burden of comorbidities, including higher rates of anxiety and HIV, despite being approximately 5.3 years younger on average than their non-LGBTQ+ individuals living with PD. These findings suggest that LGBTQ+ individuals living with PD may face disproportionate health challenges that extend beyond the age differences.

Additionally, younger participants (<65 years) reported worse physical, mental, and social health outcomes than older participants, which may be a pattern that seems counterintuitive given the progressive nature of PD. One possible explanation is that younger participants had early-onset PD or more rapidly progressing PD, which could contribute to worse health outcomes. However, we are unable to examine these aspects given that our study did not collect data on disease severity, duration, or stage.

Several other explanations should also be considered. First, survivor bias may have influenced our findings, as older participants who were able to complete a 45-min telephone survey may represent a healthier subset of older adults living with PD. Second, younger participants may have been more likely to have early-onset PD, which has been associated with distinct clinical trajectories and greater psychosocial burden (2, 23). Third, younger individual living with PD may be more likely to be balancing employment and caregiving responsibilities, creating additional stressors that could adversely affect mental and social health. Finally, differences in healthcare-seeking behavior, symptom awareness, or expectations regarding health across age groups may also explain these differences. Future longitudinal studies that include detailed clinical measures are needed to better disentangle the contributions of age, disease duration, disease severity, and social factors to these observed differences.

These disparities may reflect the cumulative effects of minority stress, barriers to accessing social and economic resources, and broader social determinants of health that may shape long-term PD-related outcomes. Consistent with our findings, studies have found that LGBTQ+ older adults face physical, mental, and social health disparities (24, 25). Although few studies have examined differences in physical and mental health between LGBTQ+ and non-LGBTQ+ individuals living with PD, research with LGBTQ+ aging populations living with similar neurodegenerative diseases, such as dementia and cognitive decline, have found greater physical and mental health disparities (26, 27). This may suggest that health disparities observed among LGBTQ+ aging populations may extend across multiple neurodegenerative diseases, including PD.

### 4.2 Sociodemographic characteristics

The LGBTQ+ group in our sample was, on average, 5.3 years younger than the non-LGBTQ+ group and differed significantly in terms of sex assigned at birth and marital status. Although age, marital status, and other sociodemographic characteristics were included as covariates in our multivariable models, residual confounding could not be ruled out and the observed disparities between LGBTQ+ and non-LGBTQ+ participants may partly reflect these underlying demographic differences rather than LGBTQ+ identity. Matching strategies (e.g., age-matching or propensity score matching) were not employed given our modest sample size, which would have limited statistical power for both the matched analysis and the broader examination of social determinants of health that was a central aim of this study. Future studies with larger, matched, or longitudinal samples are needed to more precisely disentangle the independent contribution of LGBTQ+ status from correlated demographic factors.

### 4.3 Healthcare discrimination as a driver of poor outcomes

Our study also found that LGBTQ+ individuals living with PD reported greater healthcare discrimination than non-LGBTQ+ individuals living with PD. These findings are consistent with prior research showing that LGBTQ+ individuals living with PD experience more discrimination in healthcare settings, as well as greater social and economic disadvantages, including lower income and higher rates of unemployment, compared with older cisgender heterosexual men living with PD (11). These findings likely highlight the challenges LGBTQ+ individuals may face in managing a progressive neurodegenerative disease while navigating systemic barriers that can contribute to inequitable access to healthcare and support services.

We found that healthcare discrimination was associated with moderate-to-severe loneliness, poorer health-related quality of life, and greater depressive symptoms. However, because of the cross-sectional design, these findings do not establish the temporal or causal direction of these associations. It is possible that experiences of healthcare discrimination are associated with poorer psychosocial outcomes, that individuals experiencing greater loneliness or depressive symptoms are more likely to perceive or report discrimination, or that these relationships are bidirectional. These findings align with prior research demonstrating that stigma and discrimination were associated with worse mental health outcomes, reduced quality of life, and lower overall wellbeing (28–30). There is a need for culturally responsive and inclusive healthcare that addresses discrimination, reduces stigma, and promotes mental health and quality of life among diverse populations living with PD.

### 4.4 Social and structural determinants of physical and mental health

Several social determinants of health were associated with a higher burden of PD-related physical, mental, and social health challenges. For instance, we found that younger age, identifying as a person of color, not being married/partnered, and reporting a lower income were associated with moderate to severe non-motor-related PD symptoms, loneliness, worse quality of life, and greater depressive symptoms. PD-related studies have mostly examined the role of social determinants of health on PD-related quality of life. These studies have found differences in quality of life based on age, education, gender, employment, income, marital status, social relationships/social support, and race/ethnicity (23, 31, 32).

Depression and depressive symptoms are often elevated among individuals living with PD and among those with neurodegenerative diseases (33, 34). Similar to our study, other studies have found that age, marital status, and income were associated with depression among individuals living with PD (35). Our study found that LGBTQ+ individuals living with PD reported greater loneliness than non-LGBTQ+ individuals living with PD. Greater loneliness was also reported among individuals living with PD who identified as people of color, were unmarried, and had lower incomes. Although few studies have examined determinants of loneliness among individuals living with PD, one study found that social exclusion and material deprivation (i.e., lack of economic resources) were more common among individuals living with PD and were associated with poorer quality of life and mental and physical health (36).

Effective care for persons living with PD requires moving beyond symptom management alone, toward an interdisciplinary approach that actively screens for comorbid conditions, addresses social isolation and loneliness, and provides culturally affirming care. LGBTQ+ communities remain largely absent from PD research, leaving significant gaps in our understanding of how best to support their care needs. Future research must prioritize the inclusion of LGBTQ+ persons living with in PD, with particular attention to the intersection of minority stress, chronic conditions, and social determinants of health shape disease outcomes.

## 5 Limitations of the study

While the current study revealed key factors associated with PD-related symptoms among diverse individuals living with Parkinson’s, several limitations should be considered. First, the study was cross-sectional and relied on a convenience sample. Participants were primarily recruited from Michael J. Fox Buddy Network, a free online peer support platform that connect members of the PD community. Despite our study revealing elevated rates of loneliness, these estimates may underestimate the extent of loneliness among the broader PD community, especially given many of our study participants had some level of connection to others living with PD through the Buddy Network. However, participants recruited through this network likely differ from the broader PD population in terms of their healthcare engagement, education, social support, digital literacy, community connectedness and willingness to disclose their LGBTQ+ identities. This also limits the external validity of our findings and results should be interpreted cautiously. Additionally, despite the current study revealing elevated rates of loneliness, these estimates may underestimate the extent of loneliness among the broader PD community, especially given many of our study participants had some level of connection to others living with PD through the Buddy Network. This likely limits the generalizability of our study findings to other populations living with PD.

In addition, snowball sampling may have introduced self-selection bias, as individuals who are more engaged with the PD community and more willing to disclose their identity may be overrepresented in the sample. Moreover, findings may not be fully representative of the broader LGBTQ+ and non-LGBTQ+ communities living with PD. Our relatively small sample size also limited statistical power to examine differences among LGBTQ+ and racial/ethnic subgroups. Additionally, participants generally had high levels of education and income. Given that race/ethnicity and socioeconomic status are important social determinants of health, future research should prioritize recruitment strategies that engage socioeconomically and racial/ethnically diverse populations living with PD. Additionally, this study included numerous bivariate comparisons across demographic characteristics, health conditions, and PD-related outcomes. Because the study was exploratory and intended to generate hypotheses, we did not correct for multiple comparisons (e.g., Bonferroni). As a result, the risk of Type I error was increased, and some statistically significant associations may represent chance findings rather than true underlying relationships. Our results should be interpreted with caution and considered preliminary until replicated in other studies with predetermined hypotheses. It is also important to note that the discrimination measures used in this study did not distinguish among different forms of discrimination (e.g., based on sex, race/ethnicity, gender identity, or sexual orientation), limiting our ability to determine which types of discrimination are most strongly associated with PD-related symptoms.

Finally, our survey did not collect clinician-verified or medical record-based clinical variables, such as disease duration, age at PD diagnosis, current medication regimen, levodopa equivalent daily dose, or Hoehn and Yahr disease stage. These are well-established predictors of symptom severity and functional impairment in PD, and their absence limit our ability to determine whether the disparities observed between LGBTQ+ and non-LGBTQ+ participants, as well as across other demographic characteristics, reflect differences in underlying disease severity, social determinants of health, or an interaction between these factors.

Additionally, although our study focused on *a priori* social determinants of health, variable selection for the multivariable regression models was based on those that demonstrated significant bivariate associations. Given our modest sample size, this approach was intended to produce more parsimonious models and reduce the risk of overfitting. However, we acknowledge that this data-driven strategy may have excluded clinically or theoretically important confounders that did not reach statistical significance in unadjusted analyzes and is not a substitute for a priori confounder selection based on existing evidence. Consequently, our findings may be subject to residual confounding, limiting our ability to fully disentangle the relative contributions of disease severity and social determinants of health to the observed associations, and should be interpreted accordingly.

## 6 Conclusion

This study provides important insights into the role of social determinants of health in shaping the experiences of LGBTQ+ and non-LGBTQ+ individuals living with PD. Future research should prioritize larger and more diverse samples of individuals living with PD to better understand how factors such as discrimination influence symptom burden and disease trajectories. Additional research is also needed to examine the experiences of transgender and gender-diverse individuals, bisexual and queer populations, and people aging with both PD and HIV. Finally, efforts to address and reduce structural inequities are essential for improving healthcare experiences and outcomes among diverse populations living with PD. Without such efforts, disparities in health and wellbeing will likely persist among an already vulnerable population.

## Data Availability

The raw data supporting the conclusions of this article will be made available by the authors, without undue reservation.
